# Supplementary material for: Correlates of Children’s Independent Mobility in Canada: A Multi-Site Study
Source: Int J Environ Res Public Health. 2019 Aug 10;16(16):2862. doi: 10.3390/ijerph16162862 (PMC6727085; doi:10.3390/ijerph16162862)
Supplement: Supplementary file 1 [file ijerph-16-02862-s001.pdf]

Table S5: Individual-level correlates of children's independent mobility using complete cases.

Correlate	Girls (n=931)			Boys (n=762)		
	β	95% CI		β	95% CI	
**Child Characteristics**						
Child grade level	**0.621*****	0.491	0.751	**0.596*****	0.443	0.749
Child illness	-0.281	-0.793	0.232	-0.390	-0.882	0.102
Mobile phone ownership	**0.401***	0.083	0.719	0.353	-0.059	0.765
**Household Characteristics**						
Parent age	**-0.328****	-0.562	-0.094	0.086	-0.175	0.347
Parent gender	-0.031	-0.278	0.216	-0.215	-0.519	0.088
Parent work status (not working vs working)	**0.530****	0.191	0.869	0.150	-0.224	0.524
Parent education	0.179	-0.142	0.500	-0.224	-0.625	0.176
Language spoken (English/French vs other language)	**-0.577*****	-0.873	-0.280	**-0.453****	-0.778	-0.129
Car ownership	-0.203	-0.904	0.499	0.070	-0.633	0.773
Home ownership	-0.226	-0.507	0.056	0.108	-0.229	0.446
Siblings (no sibling vs sibling(s))	0.000	-0.286	0.287	0.046	-0.279	0.370
**Parent Travel Mode to Work**						
Walk	-0.193	-0.539	0.152	0.220	-0.202	0.643
Bike	**0.478***	0.058	0.897	0.202	-0.348	0.753
Public transit	-0.204	-0.572	0.163	**-0.518***	-0.921	-0.116
Car	-0.200	-0.459	0.059	0.044	-0.278	0.366

Significant correlates are bolded: $^{1}p<.05$, $^{2}p<.01$, $^{3}p<.001$; CI: Confidence Interval; β: Unstandardized regression coefficients

Table S6: Social environment-level correlates of children's independent mobility using complete cases.

**Correlate**	**Girls (n=931)**			**Boys (n=762)**		
	β	95% CI		β	95% CI	
**Child Perceptions**						
Neighbourhood safety	**0.170****	0.047	0.294	**0.260****	0.110	0.410
**Child worried about…**						
Traffic	0.033	-0.290	0.356	-0.225	-0.595	0.144
Getting lost	-0.237	-0.576	0.102	**-0.397***	-0.769	-0.025
Bullying	0.099	-0.254	0.453	0.299	-0.111	0.709
Strangers	-0.125	-0.442	0.193	0.001	-0.314	0.316
Feeling they are not old enough to go about on their own	-0.094	-0.484	0.296	-0.285	-0.772	0.203
Not knowing what to do if someone speaks to them	-0.142	-0.454	0.169	**-0.360***	-0.710	-0.010
**Parent Perceptions**						
Most adults in the neighbourhood look out for other people's children in the area	0.024	-0.106	0.154	0.044	-0.098	0.186
People in the area make me afraid to let child play outdoors	-0.081	-0.206	0.044	-0.038	-0.176	0.099
Worried about risk of child being injured in a traffic accident	**-0.523*****	-0.679	-0.366	**-0.399*****	-0.569	-0.229
**Barriers to child walking or cycling**						
No sidewalks or bike lanes	0.002	-0.013	0.017	0.005	-0.010	0.020
Route does not have good lighting	-0.003	-0.016	0.009	-0.001	-0.020	0.017
Too much traffic around the home	0.007	-0.003	0.018	0.003	-0.017	0.023
One or more dangerous crossing	**-0.011***	-0.022	-0.001	0.002	-0.009	0.014
Unsafe due to crime (strangers, gangs, drugs)	0.002	-0.016	0.020	0.002	-0.032	0.037
Child gets bullied, teased, harassed	-0.001	-0.017	0.015	-0.014	-0.032	0.005

Significant correlates are bolded: ^{1}p<.05, ^{2}p<.01, ^{3}p<.001; CI: Confidence Interval; β: Unstandardized regression coefficients

Table S7: Geographical and area-level correlates of children's independent mobility using complete cases.

**Correlate**	**Girls (n=931)**			**Boys (n=762)**		
	β	95% CI		β	95% CI	
**Site**						
Ottawa, ON	**-1.311*****	-1.719	-0.904	**-1.081*****	-1.582	-0.580
Vancouver, BC	**-1.376*****	-1.767	-0.985	**-1.218*****	-1.691	-0.745
Trois Rivières, QC	0	.	.	0	.	.
**Urbanization**						
Urban	-0.207	-0.669	0.256	**-1.582***	-1.071	-0.002
Suburban	-0.281	-0.702	0.141	**-1.691****	-1.276	-0.264
Rural	0	.	.	0	.	.
**Walkability**						
400 m	**0.072****	0.023	0.122	-0.007	-0.065	0.051
1600 m	**-0.070***	-0.128	-0.013	0.032	-0.028	0.092
Socioeconomic status	-0.178	-0.503	0.146	-0.135	-0.528	0.258

Significant correlates are bolded: ^{1}p<.05, ^{2}p<.01, ^{3}p<.001; CI: Confidence Interval; β: Unstandardized regression coefficients
